# P-1199. Well-Balanced Dual-Targeting of Gepotidacin against Neisseria gonorrhoeae Gyrase and Topoisomerase IV in Cells and in vitro

**DOI:** 10.1093/ofid/ofaf695.1392

**Published:** 2026-01-11

**Authors:** Alexandria A Oviatt, Jessica A Collins, Jianzhong Huang, Karen Mattern, Pan Chan, Neil Osheroff

**Affiliations:** Vanderbilt University School of Medicine, Nashville, Tennessee; Vanderbilt University School of Medicine, Nashville, Tennessee; GlaxoSmithKline, Collegeville, Pennsylvania; GlaxoSmithKline, Collegeville, Pennsylvania; GlaxoSmithKline, Collegeville, Pennsylvania; Vanderbilt University School of Medicine, Nashville, Tennessee

## Abstract

**Background:**

Gonorrhea, a sexually transmitted infection caused by *Neisseria gonorrhoeae*, poses a significant public health challenge. Fluoroquinolones (FQs) once served as first-line therapy for gonorrhea, but rising resistance led to their removal from treatment guidelines. FQ resistance stems primarily from mutations in their enzyme targets, gyrase and topoisomerase IV. In response to the growing threat of antibacterial resistance, gepotidacin (Fig.), a first-in-class triazaacenaphthylene, offers a promising new treatment strategy. Gepotidacin also targets gyrase and topoisomerase IV but is chemically and mechanistically distinct from FQs. In a phase 3 clinical trial, oral gepotidacin demonstrated non-inferiority to ceftriaxone + azithromycin for uncomplicated urogenital gonorrhea with no new safety concerns [Ross et al (2025) *Lancet*].

**Figure:**
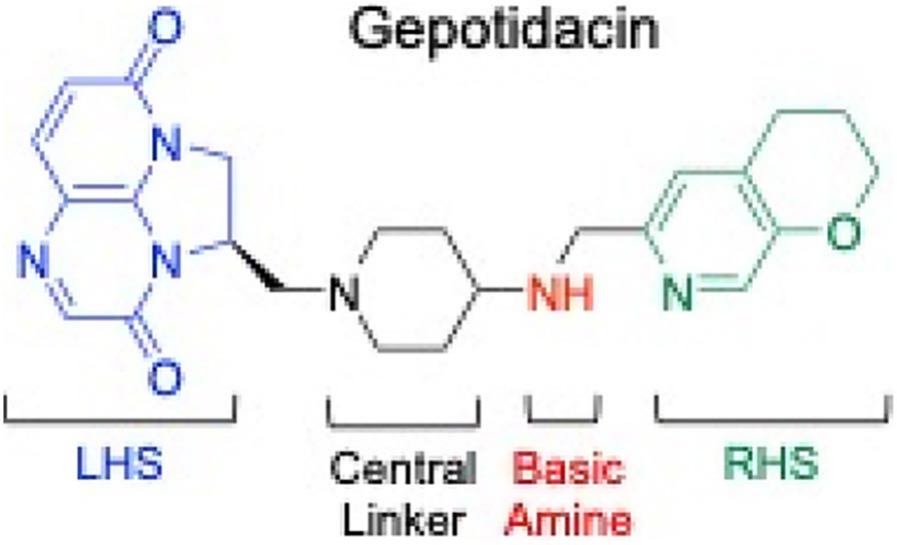

Structure of the triazaacenaphthylene gepotidacin with key pharmacophoric elements shown: left-hand side triazaacenaphthylene that pi-pi stacks with two central base pairs of the stretched DNA (LHS, blue), central linker (black), basic amine that interacts with the aspartic acid (N. gonorrhoeae GyrA D90 or ParC D86; red), and right-hand side that binds in a largely hydrophobic GyrA or ParC pocket that opens up on the dimer interface (RHS, green).

**Methods:**

Despite this success, little is known about how gepotidacin interacts with its *N. gonorrhoeae* targets. Thus, we undertook genetic and biochemical studies to determine the targeting of gepotidacin in *N. gonorrhoeae* cells and its effects on purified *N. gonorrhoeae* gyrase and topoisomerase IV.

**Results:**

Unlike FQs, which primarily target gyrase, gepotidacin displayed well-balanced dual-targeting of gyrase and topoisomerase IV in

*N. gonorrhoeae* cells. Reduced susceptibility to gepotidacin required concurrent gepotidacin target-specific mutations in both enzymes, predicting a low propensity for developing target-mediated resistance. Gepotidacin also maintained activity against cells harboring the most common FQ-resistant mutations. Consistent with its cellular dual-targeting, gepotidacin inhibited DNA supercoiling and decatenation catalyzed by purified *N. gonorrhoeae* gyrase and topoisomerase IV, respectively, at similar low µM concentrations, and induced primarily single-stranded DNA breaks by both enzymes at comparable concentrations. Mutations in key aspartic acid residues in *N. gonorrhoeae* gyrase (GyrA^D90^) and topoisomerase IV (ParC^D86^), predicted to mediate the most important gepotidacin-protein interactions, diminished the activity of gepotidacin against both enzymes.

**Conclusion:**

These insights into the targeting and mechanism of action of gepotidacin support its potential use in treating gonorrhea.

**Disclosures:**

Jianzhong Huang, PhD, GlaxoSmithKline: Employee Karen Mattern, MSc, GlaxoSmithKline: Employee Pan Chan, PhD, GlaxoSmithKline: Employee

